# Effects of Cacao Flavonoids in Long COVID-19 Patients with Chronic Fatigue: FLALOC, a Placebo-Controlled Randomized Clinical Trial

**DOI:** 10.3390/jcm15041468

**Published:** 2026-02-13

**Authors:** Levy Munguía, Selene Silva, Francisco Villarreal, Nayelli Nájera, Guillermo Ceballos

**Affiliations:** 1 Clínica de Especialidades Indianilla, Instituto de Seguridad y Servicios Sociales para los Trabajadores del Estado, Mexico City 06720, Mexico; 2 VA San Diego Health Care, San Diego, CA 92093, USA; 3 Sección de Estudios de Posgrado e Investigación, Escuela Superior de Medicina, Instituto Politécnico Nacional, Mexico City 11340, Mexico

**Keywords:** long COVID, chronic fatigue, flavanols, epicatechin

## Abstract

**Background:** In the context of long COVID, persistent fatigue is among the most prevalent symptoms that can develop after SARS-CoV-2 infection. Mitochondrial myopathy and endothelial dysfunction, which are triggers of inflammation, have emerged as prominent causes of long COVID-induced fatigue. Interestingly, the intake of flavanols, particularly (−)-epicatechin (EC), has been associated with the positive modulation of endothelial and mitochondrial structure and function. **Methods:** In this work, we conducted a randomized, double-blind, placebo-controlled clinical trial to determine whether an EC-enriched supplement (ECES) improves plasma markers of inflammation, endothelial structure, and fatigue-related endpoints in patients with long COVID-19. **Results:** The study included 46 subjects (mean age 52 years) who were instructed to consume two capsules/day for 90 days of either ECES (*n* = 23) or placebo (*n* = 23). Endpoints assessed included mean changes in plasma inflammatory markers (IL-1β, IL-6, and TNF-α) and endothelial dysfunction markers (syndecan-1), handgrip strength, fatigue scale, and quality of life (QoL). The results showed significant improvements in the ECES group for inflammatory markers, syndecan-1, and fatigue compared with the placebo group. **Conclusions:** The results yield intriguing positive findings for EC and open a new avenue for treating long COVID.

## 1. Introduction

Coronavirus disease 2019 (COVID-19) is caused by the acute respiratory syndrome coronavirus 2 (SARS-CoV-2). An immune response follows SARS-CoV-2 infection [1]. It includes increased interferons, tumor necrosis factor, bradykinin, serine proteases, and soluble thrombomodulin, and altered clot lysis times [2,3,4,5,6], all of which contribute to microvascular and thrombotic disease [7,8].

Recent reports also indicate that the coronavirus can alter mitochondrial structure and function, both directly and indirectly. Activation of the immune system, in addition to endothelial and mitochondrial dysfunction, leads to an inflammatory response that can persist for days or longer.

Approximately 10% of survivors of COVID-19 suffer from an array of symptoms that can persist for extended periods of time, referred to as long COVID [9,10,11]. A prominent and common symptom of long COVID is the development of fatigue. A myriad of causes can trigger fatigue, but its development has been strongly associated with post-viral phases of infection. Long COVID-associated fatigue has been causally linked to sustained inflammation and to endothelial and mitochondrial dysfunction.

We recently reported results from a clinical study on the relationship between COVID-19 severity and circulating syndecan-1 (a marker of endothelial glycocalyx damage) and inflammatory markers (CRP, IL-6, and TNF-α). Serum syndecan-1 levels were significantly higher in patients presenting with severe acute COVID-19 infection than in those with mild or moderate disease, indicating greater glycocalyx/endothelial damage. Markers of inflammation also increased significantly, particularly with severe disease [12].

The search for specific markers and treatments that ameliorate long COVID-associated symptoms has yielded few positive outcomes, particularly for fatigue.

Flavonoids are natural products abundant in foods such as berries and cacao. In preclinical and clinical studies, regular consumption of flavanol-enriched cacao products has been associated with improvements in markers of inflammation, vascular function, and muscle structure and function. Such effects have been attributed to (−)-epicatechin (EC), which is abundantly present in cacao beans. Recently, we reported that a flavanol-enriched cocoa supplement improves cardiometabolic status, physical performance/mobility, and quality of life (QoL) in individuals aged 60 years and older [13], with reductions in oxidative stress and inflammatory biomarkers, decreased fatigability, facilitated physical activity, and increased mobility. Using an epicatechin-enriched supplement (ECES) administered for 3 months, we also reported positive effects of daily supplementation on cardiovascular disease risk and inflammatory markers in postmenopausal women compared with placebo [14].

In the present study, we tested the hypothesis that 3-month supplementation of long COVID patients with an ECES (compared with placebo) would significantly improve measures of fatigue, quality of life (QOL), and serum markers of inflammation and glycocalyx damage.

## 2. Materials and Methods

The National Research and Ethics Committee (ISSSTE) approved the study (309.2022), and it was registered on ClinicalTrials.gov (NCT06064838, posted on 2 October 2023) in accordance with trial reporting requirements. The present study is a randomized, double-blind study, conducted at the Specialty Clinic Indianilla of the Institute for Social Security and Services for State Workers (ISSSTE) in Mexico City, following registration on ClinicalTrials.gov, from October 2023 to June 2024.

### 2.1. Study Design and Randomization

**Figure 1:**
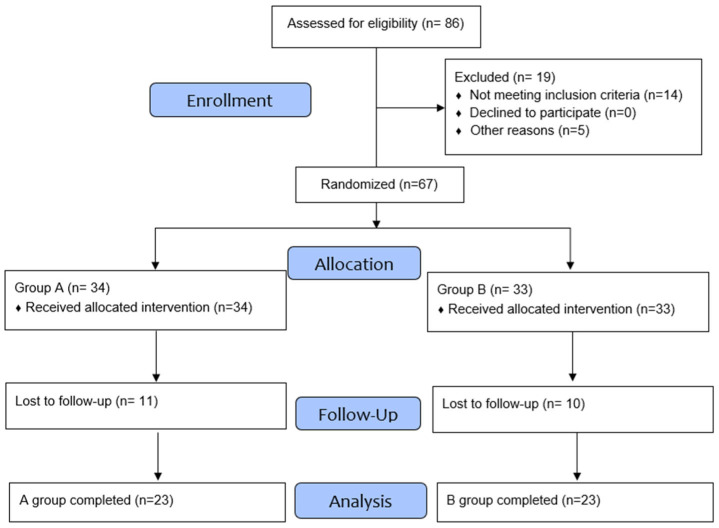
Flow diagram for the enrollment of study participants.

Participants were randomized into two groups, ECES and placebo, in a 1:1 ratio using an online research randomizer software (https://www.randomizer.org) (Figure 1). The experimental group received non-commercially available capsules containing 500 mg of ECES (comprising cacao husk flour and 40 mg of free epicatechin) twice daily. The product was prepared in a certified GMP facility (Grupo Pronatur, Monterrey NL, Mexico). The placebo group received one capsule with 500 mg of excipients twice daily. Supplementation lasted for 90 days.

Participants were included in the study if they met the following criteria: post-COVID-19 patients with at least 6 months after symptom onset and chronic fatigue. Participants were excluded from the study if they had severe comorbidities, such as chronic liver disease or chronic kidney disease; had allergies or intolerances to the listed ingredients; were pregnant or lactating; or had a BMI greater than 35. Ultimately, 46 post-COVID-19 patients who came to the clinic for chronic fatigue symptoms completed the study (23 per group). After an initial assessment, follow-up was performed every 30 days. Figure 1 provides a detailed flowchart of patient screening, inclusion, randomization, and study follow-up.

### 2.2. Fatigue

The fatigue level was determined using the fatigue numeric rating scale (NRS), which is a single-item, horizontal scale ranging from 0 to 10, with zero representing ‘no fatigue’ and ten representing ‘as bad as you can imagine’. Patients were asked to rate their fatigue by selecting the number that best describes their worst level of fatigue over the past 24 h.

### 2.3. Quality of Life (QoL)

Quality of life was evaluated using the EQ-5D questionnaire, a self-administered health index that explores five domains with five levels of severity (mobility, self-care, activities of daily living, pain/discomfort and anxiety/depression) with a scale from one (no problems) to five (problems/external impossibility) and a visual analog scale (a self-report tool for measuring pain intensity, health states, or other symptoms on a continuous line) to measure the quality of life, graded 0 (worst) to 100 (best) for the perception of wellbeing.

### 2.4. Handgrip Strength

Using the dominant hand, strength was measured with a digital handgrip dynamometer, with the average of three consecutive maximal-effort grips recorded. The highest value measured was considered the patient’s representative handgrip strength.

### 2.5. Pro-Inflammatory Cytokines and Endothelial Damage Biomarkers

Blood (10 mL) was drawn at the beginning (pre-treatment) and 90 days into the trial. Serum was isolated and frozen at −80 °C until assayed. Serum samples were assayed for biomarkers of inflammation (IL-1β, IL-6, and TNF-α) and for syndecan-1, a marker of endothelial glycocalyx damage. Biomarkers were measured using commercially available kits following the manufacturer’s instructions. IL-1 β was determined using the Human IL-1 beta ELISA Kit (Abcam, catalog # ab214025, Cambridge, UK). IL-6 was determined using the Human IL-6 ELISA Kit (Abcam, catalog # ab178013, Cambridge, UK). TNF-α was determined using the Human TNF alpha ELISA Kit (Abcam, catalog # AB181421, Cambridge, UK). Syndecan-1 (SDC1) was measured using the Human Syndecan-1 ELISA Kit (Abcam, catalog # AB46506, Cambridge, UK). Each sample was assessed in duplicate and averaged, and the results were expressed as mean ± SD. Hemolyzed sera were discarded due to interference with the detection method.

### 2.6. Statistical Analysis

Variables are reported as mean ± standard deviation (SD) or percentages when appropriate. Data were plotted, and statistical analyses (simple or paired *t*-tests) were performed using Prism version 10.0 for Mac (GraphPad, CA, USA). A *p*-value < 0.05 was considered statistically significant.

## 3. Results

The average age of recruited patients was 52.72 ± 8.19 years, with a mean number of 1.8 ± 1.2 COVID-19 infections (Table 1). Males constituted 76% of the sample, and 24% were female.

The primary symptoms reported by patients are listed in Table 2.

**Figure 2:**
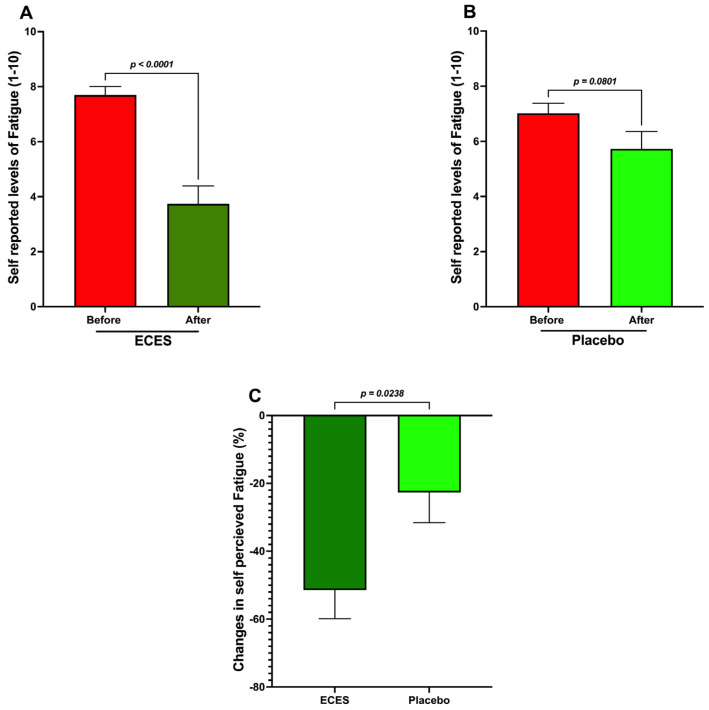
Analysis of the effects induced by three months of treatment (**A**) with an ECES and placebo (**B**). Comparison of the percentage change in self-reported fatigue between an ECES and a placebo (**C**). Data are presented as mean ± SD. Data were analyzed using paired (**A**,**B**) or simple (**C**) *t*-tests, with *p*-values reported.

Interestingly, self-reported fatigue levels decreased by a significant 51% in the ECES-treated group (Figure 2A) and by a non-significant 22.6% in the placebo group (Figure 2B). The comparison of the treatment-induced percentage changes showed a significant difference between groups (Figure 2C).

**Figure 3:**
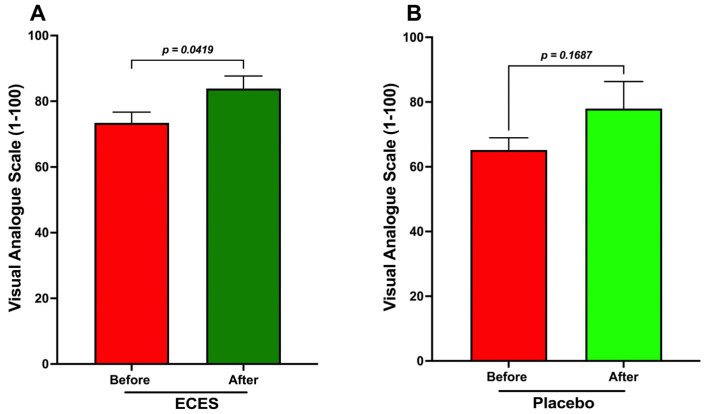
Analysis of the effects induced by three months of treatment (**A**) with an ECES and placebo (**B**). Data are presented as means ± SD. Data were analyzed using a paired *t*-test, with *p*-values shown.

The analysis of quality of life (QoL) using the visual analog scale showed that treatment with the ECES increased the quality of life as compared with the placebo-treated group, where a non-significant change was noted (Figure 3).

**Figure 4:**
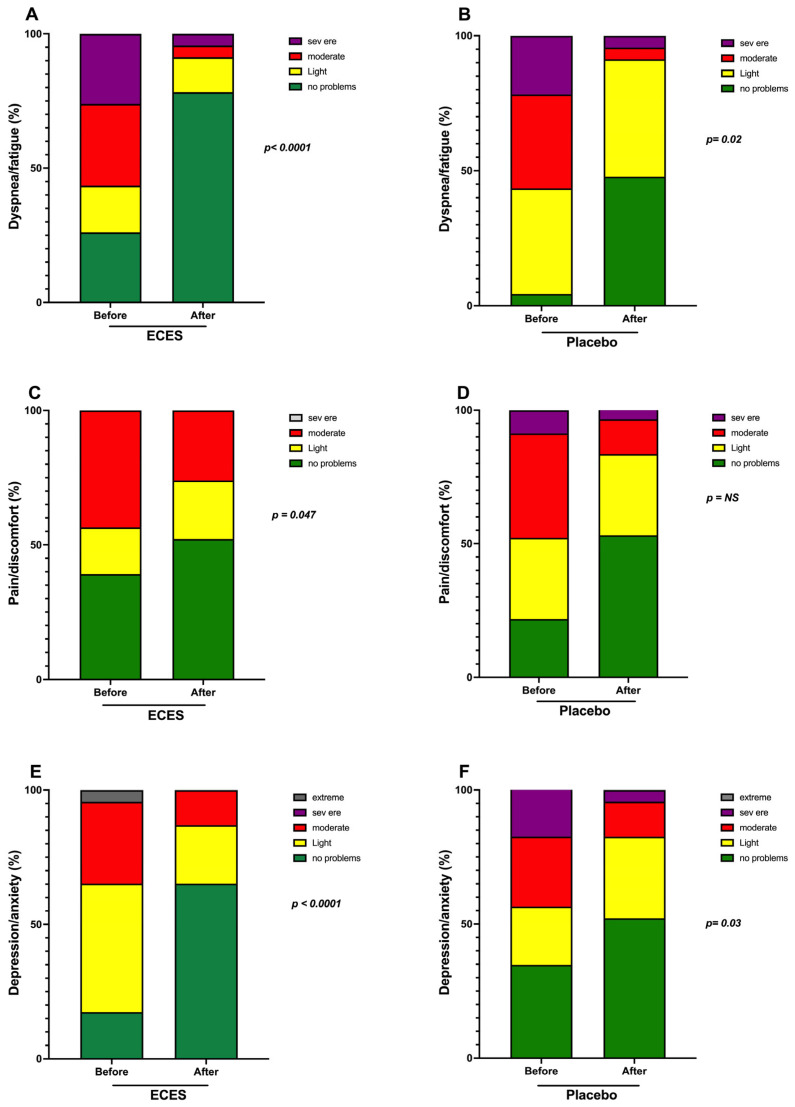
Sub-analysis of the visual analog scale showing the effects induced by three months of treatment (**A**) with an ECES and placebo (**B**) in dyspnea/fatigue. Pain/discomfort (**C**) with an ECES and placebo (**D**) and depression/anxiety with an ECES (**E**) and placebo (**F**). Data are presented as mean ± SD. Data were analyzed using a paired *t*-test, and *p*-values are reported.

A sub-analysis of the visual analog scale revealed significant improvements in dyspnea/fatigue for both groups. However, improvements were larger in the ECES group vs. placebo (Figure 4A,B). Pain/discomfort was significantly lower in the ECES-treated group vs. placebo (Figure 4C,D), and depression/anxiety were significantly lower in both groups (Figure 4E,F).

**Figure 5:**
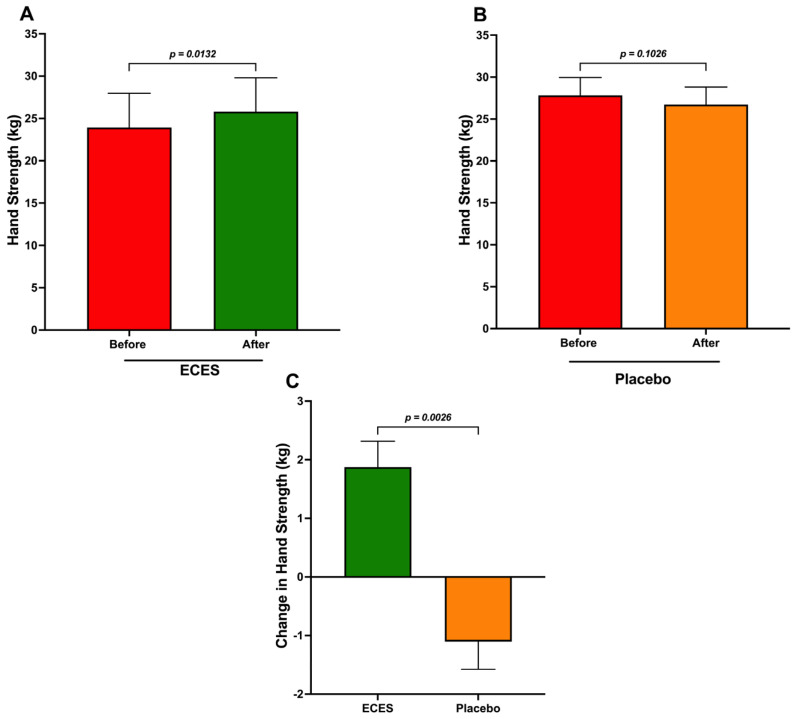
Analysis of the effects induced by three months of treatment (**A**) with an ECES and placebo (**B**) on hand strength. Panel (**C**) reports absolute differences between the ECES and the placebo groups in men. Data are presented as mean ± SD. Data were analyzed using a paired *t*-test. Statistical significance was considered when *p* < 0.05.

On the other hand, handgrip strength was assessed separately in men and women, as it differs between genders. In men, treatment with the ECES increased strength in the dominant hand (Figure 5A), whereas the placebo group showed a non-significant decrease in strength (Figure 5B). The comparison of changes between both groups also yielded a significant difference (Figure 5C).

**Figure 6:**
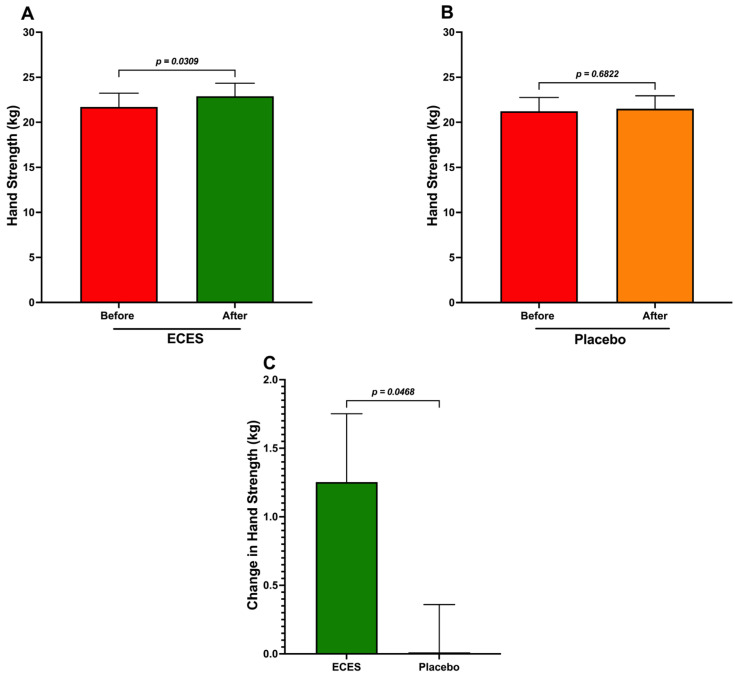
Analysis of the effects induced by three months of treatment (**A**) with an ECES and placebo (**B**) on hand strength. Panel (**C**) reports absolute differences between the ECES and the placebo groups in women. Data are presented as mean ± SD. Data were analyzed using a paired *t*-test, and the *p*-value is reported.

In women, treatment with the ECES induced a significant increase in strength in the dominant hand (Figure 6A), whereas the placebo group showed no change (Figure 6B). The comparison of changes between the two groups also showed a significant difference (Figure 6C).

**Figure 7:**
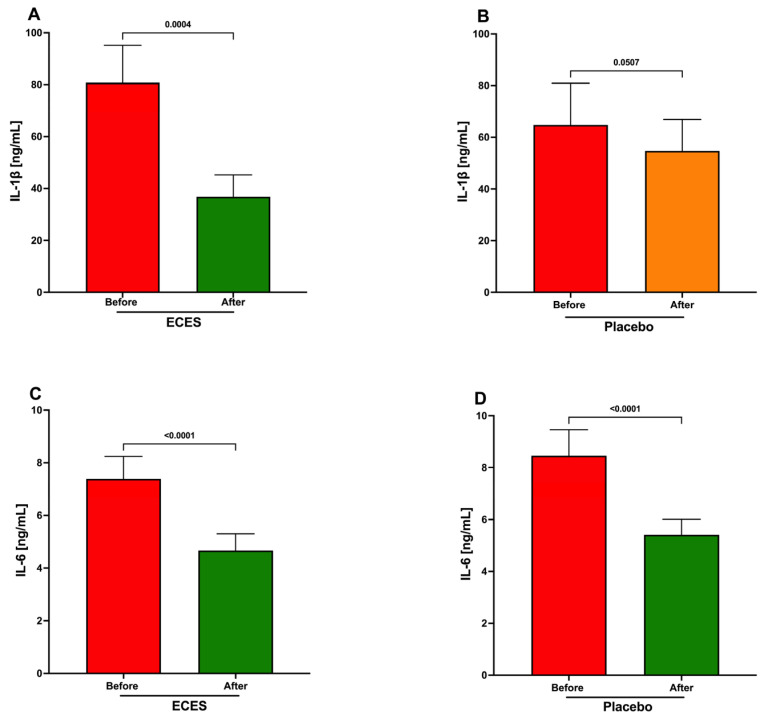
Analysis of the effects induced by three months of treatment with an ECES or placebo on serum levels of IL-1B (**A**,**B**) and IL-6 (**C**,**D**). Data are presented as mean ± SD. Data were analyzed using a paired *t*-test, with *p*-values reported.

Analysis of serum pro-inflammatory cytokine levels shows a significant decrease in IL-1β and IL-6 in the ECES-treated group (Figure 7A,C). IL-1β in the placebo group decreased but did not reach significance (Figure 7B). IL-6 also significantly decreases in the placebo group (Figure 7D).

**Figure 8:**
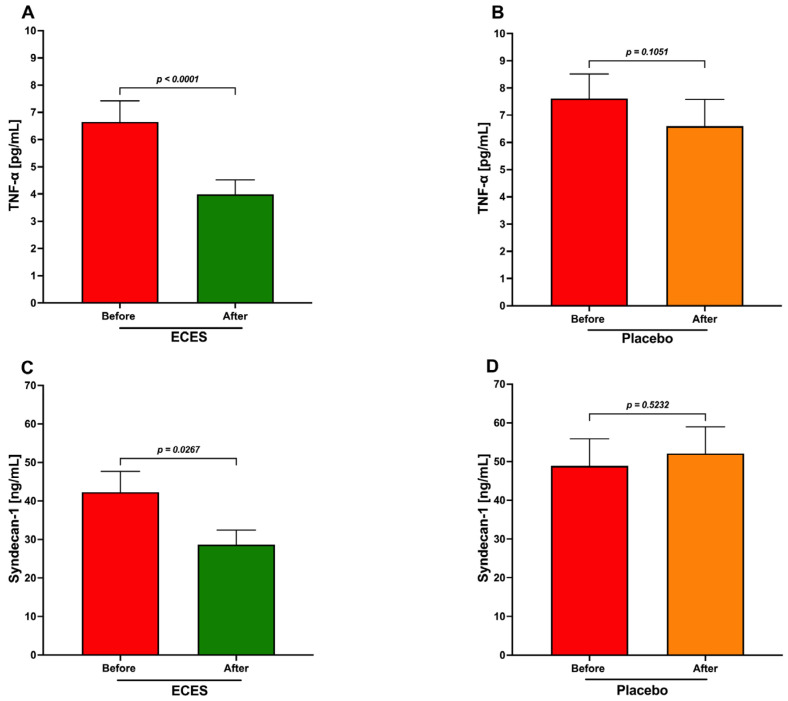
Analysis of the effects induced by three months of treatment with an ECES or placebo (**A**,**B**) on serum levels of TNF-α and blood levels. Panels (**C**,**D**) of syndecan-1 in both groups. Data are presented as mean ± SD. Data were analyzed using a paired *t*-test, with *p*-values reported.

TNF-α analysis shows a significant decrease in the ECES-treated group (Figure 8A), whereas the placebo group shows a nonsignificant reduction (Figure 8B). The analysis of syndecan-1 levels also indicates a significant decrease in the ECES-treated group (Figure 8C) and a nonsignificant modest increase in the placebo group.

## 4. Discussion

Unique results from this clinical trial demonstrate that 3-month supplementation with a flavanol-enriched product significantly reduces perceived fatigue and QOL in adults with long COVID. Fatigue reduction was accompanied by increased handgrip strength.

Underlying improvements in ECES-treated patients were a result of significant reductions in markers of inflammation and endothelial glycocalyx damage.

These findings support the hypothesis that increasing NO bioavailability [15], suppressing ROS formation [16], and modulating cell signaling pathways that protect glycocalyx integrity [17] through the synergistic effects of flavonoid metabolites ameliorate post-acute COVID-19 sequelae.

Handgrip strength (HGS) is a well-founded measurement of muscle strength and a valid predictor of disability, morbidity, and mortality [18]. In patients followed up 120 days after COVID-19 hospitalization, low HGS was associated with worse functional outcomes in long COVID [19]. In a cohort of adults, 1 year after COVID-19 hospitalization, sequelae affecting postural balance, mobility, and muscle strength were observed [20].

In our study, 1/3 of participants had low baseline handgrip strength. Those who received the epicatechin-enriched supplement experienced a significant increase in HGS, while the placebo group showed no difference. Indeed, (−)-epicatechin, both alone and in combination with other flavonoids, shows promise for preserving muscle mass and function by influencing key anabolic and catabolic pathways [21].

Numerous studies have shown that chronic fatigue is one of the most prevalent symptoms of long COVID and the one that has the most significant impact on personal and social lives [22].

Studies with flavonoids have reported beneficial effects improving the fatigue of subjects with chronic fatigue syndrome [23] or reducing associated mood features, such as depression and anxiety [24]. Our study reported a 50% reduction in fatigue in the ECES-treated group. This finding aligns with evidence suggesting that epicatechin can enhance muscle performance by modulating mitochondrial biogenesis and stimulating AKT/mTOR signaling [25], as well as by enhancing capillary angiogenesis, which is essential for skeletal muscle oxygen metabolism [26].

Improving vascular function in COVID-19 disease is essential, as the vascular endothelium regulates vasomotion, vascular permeability, inflammation, and oxidative stress [27]. Growing evidence suggests that SARS-CoV-2 acts as an endothelial disease, disrupting the glycocalyx, causing hyperpermeability, and promoting hypercoagulability. Patients with chronic endothelial dysfunction caused by cardiovascular and metabolic diseases exhibit the worst COVID-19 symptoms [28].

It has been suggested that flavonoids improve endothelial function by increasing NO bioavailability and modulating vasoactive factors such as angiotensin-converting enzyme (ACE) and endothelin-1 [29]. In our study, the significant reduction in SDC-1 levels suggests that flavonoids may be a therapeutic approach to ameliorate severe glycocalyx degradation and endothelial injury.

The exact mechanisms for the cardiovascular sequelae of long COVID remain unclear, but it is suggested that an enhanced B and T cell-mediated immune response plays a key role. Additionally, the presence of autoantibodies (e.g., anti-cardiolipin, anti-apolipoprotein A-1) and prolonged elevations of pro-inflammatory cytokines, such as TNF-α, IL-1, and IL-6, which are arrhythmogenic, are observed. There is a clinical need to identify a blood biomarker closely associated with long COVID to facilitate earlier diagnosis and potentially targeted therapy [30].

In addition, our results showed that levels of cytokines implicated in triggering immunopathological reactions in the acute phase of COVID-19, such as IL-6 and TNF-α, which persist as molecular signatures of long COVID [30], improved with ECES treatment. Altogether, ECES-induced changes lead to a reduction in perceived fatigue, thereby improving QoL.

Due to their potent anti-inflammatory, antioxidant, and immunomodulatory properties, flavonoids can help mitigate the chronic complications arising from persistent immune activation [31,32,33].

To date, there is an ongoing effort to identify effective treatments for long COVID, with more than 350 registered clinical trials investigating the efficacy of interventions such as exercise, rehabilitation, behavioral therapy, and pharmacological agents; however, results remain inconclusive [34]. In this manner, the results reported in this work provide a nutraceutical and potentially pharmaceutical alternative for the treatment of long COVID.

This work has limitations: The number of patients included was low, and the study was monocenter; in addition, the biomarkers were assessed only in patients, not in healthy individuals, thereby precluding comparisons of the magnitude of COVID-induced increase in those levels. We also did not explore the influence of disease duration on the cacao flavonoids-induced positive effects. These facts require further assessment in future studies. However, the results were statistically significant, and the trial was double-blind and placebo-controlled, which strengthens the findings.

Notwithstanding, further studies with larger populations and a multicentric design are required to confirm these promising findings. Although handgrips were used to assess muscle strength, additional tests are needed.

## 5. Conclusions

Chronic fatigue, as a major symptom of long COVID disease, has negative consequences that affect personal and social lives. Cacao flavonoid supplementation improved muscle strength, endothelial function, and fatigue in adults with long COVID. Therefore, it may be proposed as a therapeutic approach to restore physical performance in people with long COVID.

## Figures and Tables

**Table 1:** Initial data.

	All (Mean ± SD)	Placebo (Mean ± SD)	ECES (Mean ± SD)	*p*
Age (years)	52.72 ± 8.19	52.87 ± 10.6	55.35 ± 7.04	NS
N (m/f)	46 (35/11)	23 (18/5)	23 (17/6)	NS
Number of COVID-19 infections	1.87 ± 1.2	1.78 ± 1.21	1.52 ± 0.99	NS
Time since symptoms onset (days)	15.4 ± 9.4	15.2 ± 10	15.6 ± 8.9	NS

**Table 2:** Initial major symptoms.

	All (%)	Placebo (%)	ECES (%)	*p*
Headache	36/46 (73.9)	17/23 (73.9)	17/23 (73.9)	NS
Cough	23/46 (50)	11/23 (47.83)	12/23 (53.17)	NS
Shortness of breath	36/46 (78.3)	16/23 (69.57)	20/23 (86.96)	NS
Fatigue	46/46 (100)	23/23 (100)	23/23 (100)	NS
Persistent muscle pain	32/46 (69.6)	16/23 (69.56)	16/23 (69.56)	NS
Joint pain	34/46 (73.9)	18/23 (78.26)	16/23 (69.56)	NS
Sleepiness	32/46 (69.6)	16/23 (69.56)	16/23 (69.56)	NS
Confusion/lack of concentration	35/46 (76.1)	15/23 (62.22)	20/23 (86.96)	NS

## Data Availability

Data is contained within the article.
